# Empowering Healthcare Workers in Oman Through Knowledge, Attitudes, and Practices Toward Needlestick and Sharps Injury (NSI) Prevention

**DOI:** 10.7759/cureus.90942

**Published:** 2025-08-25

**Authors:** Mahmood J Al Yaqoubi, Dayana Hazwani Mohd Suadi Nata, Nur Zakiah Mohd Saat, Putri Anis Syahira Mohamad Jamil

**Affiliations:** 1 Environmental Health and Industrial Safety Programme, Center for Toxicology and Health Risk Studies (CORE), Faculty of Health Sciences, Universiti Kebangsaan Malaysia, Kuala Lumpur, MYS; 2 Programme of Biomedical Sciences, Centre for Community Health Studies (ReaCH), Faculty of Health Sciences, Universiti Kebangsaan Malaysia, Kuala Lumpur, MYS

**Keywords:** healthcare workers, injection safety measure, knowledge attitudes and practices, needlestick and sharps injuries, occupational exposure

## Abstract

Introduction

Needlestick and sharps injuries (NSIs) remain a significant occupational hazard among healthcare workers (HCWs), contributing to the risk of serious bloodborne infections and psychological distress. This study looks at how much HCWs know about preventing NSIs, their attitudes toward it, and what they do to stay safe, while also examining how their background and job affect their safety practices and the chances of getting injured.

Methods

A cross-sectional survey was conducted among 188 HCWs at Diwan Health Complex, Oman, using a validated structured questionnaire. Of the 240 HCWs approached, 188 responded (response rate: 78.3%). Sample size was calculated using a 50% prevalence assumption to ensure maximum variability and adequate precision.

Results

The mean knowledge score was 16.5 ± 2.83 (maximum: 20), reflecting good awareness overall. However, only 25% knew that soap and water should be used immediately after an NSI, and 60.6% were unaware that no post-exposure prophylaxis exists for hepatitis C. About one-third of HCWs (34.2%) reported at least one NSI. Logistic regression showed that higher knowledge scores (OR = 1.22, 95% CI = 1.05-1.43, p = 0.013) and male gender (OR = 1.83, 95% CI = 1.01-3.32, p = 0.045) significantly predicted NSI occurrence.

Conclusions

Despite adequate knowledge, critical misconceptions and unsafe practices persist, highlighting a “know-do” gap. These findings underscore the urgent need for competency-based interventions and institutional accountability. A nationwide NSI prevention training module, incorporating scenario-based education, mandatory reporting, and adoption of safety-engineered devices, is strongly recommended to reduce occupational risks and protect HCWs.

## Introduction

Needlestick and sharps injuries (NSIs) remain one of the most serious occupational hazards for healthcare workers (HCWs) worldwide, as they are a direct source of exposure to bloodborne pathogens such as hepatitis B virus (HBV), hepatitis C virus (HCV), and HIV [1-3]. Each year, WHO estimates that more than two million HCWs are exposed to NSIs, underscoring the global significance of preventive measures [2,4]. Despite increasing awareness, underreporting continues to obscure the true burden of NSIs, largely due to stigma, fear of administrative repercussions, or the perception that reporting systems are ineffective [5].

The occurrence of NSIs is closely linked to inadequate training, insufficient awareness of risks, and the persistence of unsafe practices such as needle recapping, improper disposal, and inattentive handling of sharp instruments [6,7]. Although the introduction of safety-engineered devices and structured training programs has been shown to reduce injury rates, such interventions cannot succeed in isolation. A sustainable reduction in NSIs requires addressing systemic barriers, including gaps in institutional policies, weak enforcement of safety protocols, and the absence of reliable monitoring and feedback mechanisms [8,9].

The risk of NSIs is particularly pronounced in resource-constrained settings where healthcare personnel manage heavy patient loads, multitask across procedures, and face workforce shortages. In Oman, these challenges are compounded by a multicultural workforce, evolving occupational health infrastructure, and sociocultural barriers that contribute to underreporting [10,11]. Understanding how HCWs in Oman perceive and practice NSI prevention is therefore crucial in guiding context-specific strategies. Evidence from other countries suggests that demographic and occupational characteristics, such as age, gender, work experience, and job designation, significantly influence knowledge, attitudes, and practices (KAP) levels related to NSI prevention [12,13]. Younger or less experienced HCWs may be especially vulnerable due to limited exposure to safety training, while nurses, who represent a large proportion of the healthcare workforce, are at higher risk because of their frequent use of sharps in clinical procedures [14-16].

However, there is a lack of in-depth research in Oman that specifically examines HCWs’ KAP toward NSI prevention. Existing studies in the region have largely focused on general occupational safety or infection control, without systematically addressing the behavioral, demographic, and institutional factors that shape NSI prevention practices. This creates a critical evidence gap, as Oman’s unique healthcare environment, marked by high patient-to-staff ratios, diverse cultural backgrounds among HCWs, and underdeveloped reporting mechanisms, may influence both the occurrence of NSIs and the likelihood of their reporting. Addressing this research gap is essential to inform targeted interventions, strengthen institutional policies, and support the implementation of context-specific occupational health strategies.

The primary objective of this study was to assess HCWs’ KAP regarding NSI prevention. The secondary objective was to examine behavioral and systemic factors, including demographic characteristics and institutional influences, that contribute to safety gaps and increase NSI risk. By addressing both individual-level practices and broader organizational determinants, the findings are expected to guide the development of context-specific strategies to strengthen compliance with safe injection practices, reduce NSI incidence, and foster a robust safety culture in healthcare systems.

## Materials and methods

Study design

This cross-sectional study was conducted at the tertiary healthcare facility in Oman. The study aimed to evaluate HCWs’ KAP regarding the prevention of NSIs.

Study population and sampling

The study population consisted of doctors, nurses, and allied health professionals directly involved in patient care. Stratified random sampling was applied to ensure balanced representation of professional roles, given their differing levels of exposure to sharps and safety training opportunities. Inclusion criteria required a minimum of one year of clinical experience.

The required sample size of 278 participants was calculated using Cochran’s formula, based on an assumed prevalence of 50%, a 95% confidence level, and a 5% margin of error. This conservative prevalence estimate was selected to maximize the sample size and ensure adequate statistical power, particularly in the absence of previous prevalence data from Oman. In practice, 240 HCWs were approached, and 188 completed the questionnaire, yielding a response rate of 78.3%. The shortfall relative to the calculated target was due to limited staff availability during the data collection period and the exclusion of incomplete or partially filled responses.

Data collection instrument

A structured and validated self-administered questionnaire was used to collect data, adapted from a previously published instrument [4]. The tool consisted of four main sections: demographic and occupational characteristics, knowledge domain (10 items), attitude domain (eight items), and practice domain (seven items).

Items were presented in multiple-choice and Likert-scale formats. Content validity was established through expert review by three specialists in occupational safety and infection control, and the Content Validity Index exceeded the recommended threshold of 0.80. A pilot test with 30 HCWs was conducted to assess clarity and face validity; these respondents were excluded from the final dataset. Internal consistency was satisfactory, with Cronbach’s alpha = 0.81.

Scoring of KAP domains: for the knowledge domain, correct responses were scored as “1” and incorrect or “don’t know” responses as “0,” yielding a possible range of 0-10. The attitude domain used a five-point Likert scale (1 = strongly disagree to 5 = agree), with negatively worded items reverse-coded; the total score ranged from 8 to 40, where higher scores indicated more positive attitudes. For the practice domain, safe practices were scored as “1” and unsafe practices as “0,” giving a possible range of 0-7. Higher scores across domains indicated better knowledge, more favorable attitudes, and safer practices.

Ethical considerations

Ethical approval was obtained from the Universiti Kebangsaan Malaysia Research Ethics Secretariat (approval number JEP-2024-1108). Informed written consent was obtained from all participants, and confidentiality was strictly maintained throughout the study.

Statistical analysis

The data was analyzed using IBM SPSS Statistics for Windows, Version 26.0 (Released 2018; IBM Corp., Armonk, NY, USA). Descriptive statistics (mean, standard deviation, frequency, and percentage) were used to summarize demographic characteristics and KAP scores. Group comparisons were performed using independent t-tests and one-way ANOVA, followed by post hoc analysis where appropriate. Pearson’s correlation was used to assess relationships among KAP domains. Predictors of self-reported NSIs were examined using binary logistic regression analysis. Both crude and adjusted models were presented, reporting regression coefficients (B values), standardized beta coefficients (β), ORs with 95% CIs, and p-values. Statistical significance was set at p < 0.05.

## Results

A total of 240 HCWs were invited to participate. Of these, 188 provided complete responses, while 52 questionnaires were excluded due to incomplete data, yielding a final response rate of 78.3%.

Demographics characteristics

A total of 188 HCWs participated in the study. The mean age of respondents was 39.67 years (SD = 7.02). The largest age group was 36-45 years old (n = 92, 48.9%), followed by those under 35 years old (n = 62, 33.0%). Female participants comprised 57.4% of the sample. Most respondents were Omani nationals (n = 128, 68.1%). Regarding educational attainment, over half (n = 98, 52.1%) held a bachelor’s degree, and 48 (25.5%) had a master’s diploma or higher. The demographic distribution is presented in Table 1.

**Table 1:** Demographic characteristics of HCWs HCW, healthcare worker

Demographic	n (%)	Mean ± SD	95% CI for mean
Age (years)		39.67 ± 7.02	38.65-40.69
<35	62 (33.0)		
36-45	92 (48.9)		
>46	34 (18.1)		
Gender			
Male	80 (42.6)		
Female	108 (57.4)		
Nationality			
Omani	128 (68.1)		
Non-Omani	60 (31.9)		
Education			
Secondary education	10 (5.3)		
Assistant degree	32 (17.0)		
Bachelor’s degree	98 (52.1)		
Master’s degree	48 (25.5)		

KAP toward NSIs prevention

The overall mean knowledge score was 16.5 ± 2.83 (maximum: 20), reflecting generally adequate awareness among HCWs. However, detailed domain-level analysis revealed key gaps. In the prevention domain (M = 3.63, SD = 1.08), 48.9% (n = 92) of respondents mistakenly agreed that recapping needles is acceptable, despite showing high awareness. The transmission domain (M = 3.20, SD = 1.03) showed that 67% of participants (n = 126) correctly recognized the HBV as more transmissible than HIV via NSIs. For post-exposure management (M = 3.30, SD = 1.27), knowledge was moderate; only 25% (n = 47) knew that soap and water should be used after NSI, while 60.6% (n = 114) were unaware that no post-exposure prophylaxis (PEP) exists for hepatitis C. A summary of KAP scores and related insights is provided in Table 2.

**Table 2:** Summary of knowledge domains (N = 188) p < 0.01 = significant (^*^) HBV, hepatitis B virus; HCV, hepatitis C virus; PEP, post-exposure prophylaxis

Domain	Mean (SD)	Key insight
Prevention	3.63 (1.08)	48.9% (n = 92) incorrectly agreed that recapping needles is acceptable.
Transmission	3.20 (1.03)	67% (n = 126) correctly identified HBV as more transmissible than HIV.
Post-exposure management	3.30 (1.27)	(a) Only 25% (n = 47) knew that soap and water were the correct first responses. (b) A total of 60.6% (n = 114) were unaware that there is no PEP available for HCV.
Variables	Correlation (r)	p-Value
Knowledge-attitude	0.305	<0.01^*^
Knowledge-practice	0.289	<0.01^*^
Attitude-practice	0.264	<0.01^*^

In terms of attitudes (M = 6.30, SD = 1.55), 88.8% of respondents (n = 167) agreed that increased workloads heighten NSI’s risk, suggesting a favorable perception of institutional and situational contributors to injuries. The practice domain (M = 4.10, SD = 1.12) highlighted that although most HCWs report using gloves, many admitted to inconsistent adherence with needle disposal protocols and failure to report NSIs, citing fear of blame or lack of follow-up.

**Figure 1:**
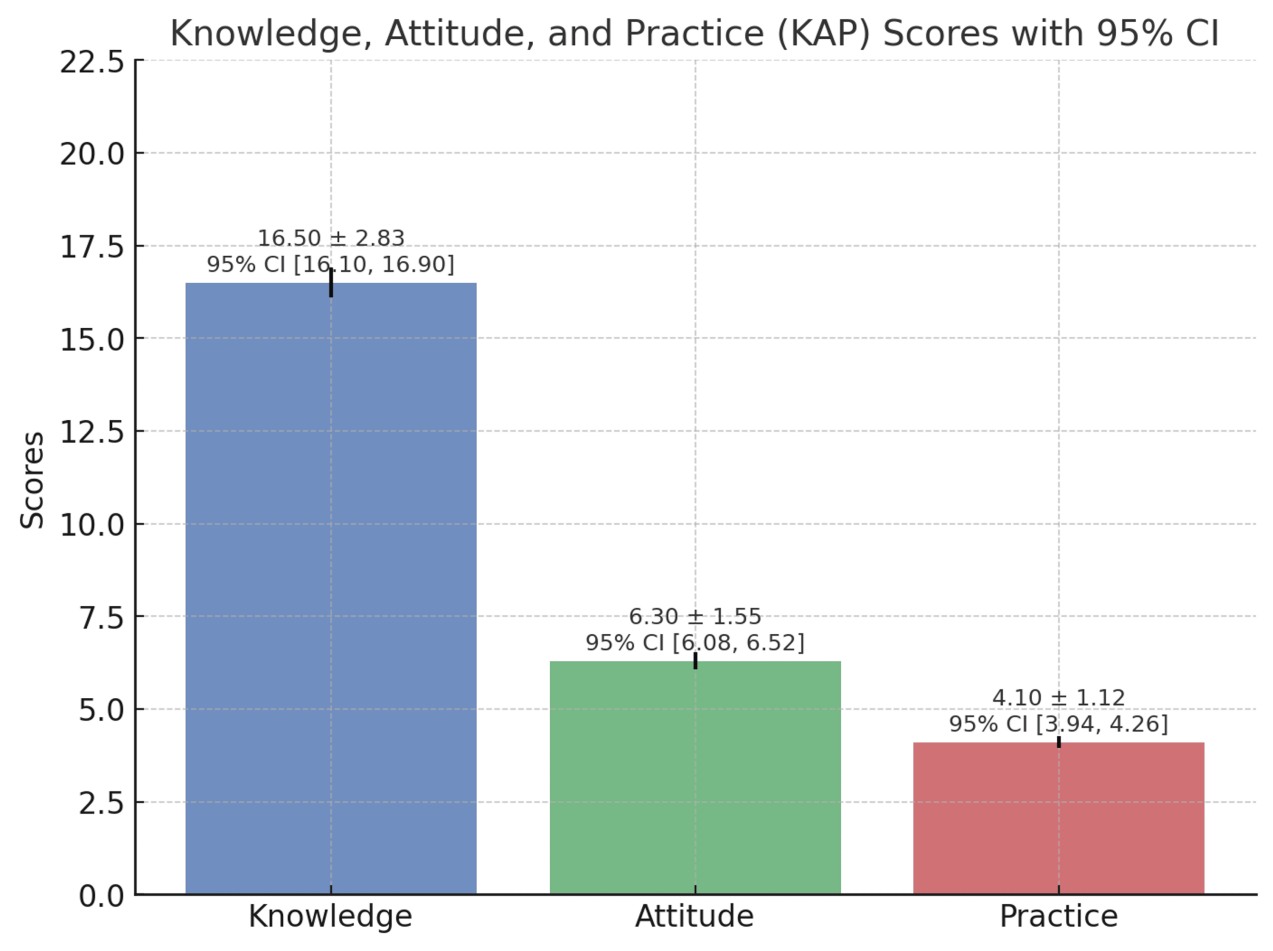
Comparison of mean scores between KAP domains KAP, knowledge, attitude, and practice

Figure 1 displays the comparison of mean scores between KAP domains.

Correlation analysis between KAP variables

The Pearson correlation analysis was performed to explore the interrelationships among KAP domains related to NSI prevention among HCWs. As presented in Table 3, all domain correlations were positive and statistically significant at the p < 0.01 level, indicating that improvements in one domain may be associated with enhancements in the others. In particular, there was a moderate positive link between knowledge and attitude (r = 0.305, p < 0.01), meaning that when HCWs know more about the risks and ways to prevent needlestick injuries, they usually have better attitudes toward safety practices. A slightly lower yet significant correlation was found between knowledge and practice (r = 0.289, p < 0.01), indicating that higher knowledge levels were associated with better compliance in safe injection and sharps handling practices. This finding is particularly important, given that translating knowledge into consistent and safe clinical behavior remains a critical challenge in healthcare settings. Additionally, attitude and practice also showed a positive association (r = 0.264, p < 0.01), reinforcing the hypothesis that positive safety attitudes may facilitate the adoption of protective practices. This correlation was the weakest among the three, but it remains statistically meaningful and points out the role of motivational and perceptual factors in shaping real-world behavior. Overall, the results from this correlation matrix provide empirical support for the interconnected nature of KAP elements in NSI prevention and underscore the importance of integrated interventions targeting cognitive, affective, and behavioral domains to drive systemic improvements in occupational safety.

**Table 3:** Correlation analysis between KAP domain variables p < 0.01 = significant (^*^) KAP, knowledge, attitude, and practice

Variables	Correlation (r)	p-Value
Knowledge-attitude	0.305	<0.01^*^
Knowledge-practice	0.289	<0.01^*^
Attitude-practice	0.264	<0.01*

NSI occurrence and logistic regression analysis

Out of the 188 HCWs surveyed, 64 (34.2%) reported experiencing at least one needlestick or sharp injury (NSI) during their professional practice, while 124 (65.8%) reported no history of such incidents. This notable incidence rate highlights the persistent occupational hazards faced by healthcare professionals and emphasizes the need for ongoing training and systemic prevention strategies in healthcare settings.

To identify predictors of NSI occurrence, a binary logistic regression analysis was conducted using knowledge scores and gender as independent variables. The overall model was statistically significant, χ²(2) = 8.92, p = 0.012, indicating that the predictors effectively differentiated between respondents who had and had not experienced NSIs. As presented in Table 4, both knowledge score and gender were significant predictors. HCWs with higher knowledge scores were more likely to report NSIs, with a 22% increase in the chances of reporting an NSI for each point increase in their knowledge score. Additionally, being male was significantly associated with increased odds of reporting an NSI (B = 0.605, p = 0.045), with an OR of 1.83 (95% CI: 1.01-3.27), suggesting that male HCWs were nearly twice as likely to experience or report NSIs compared to their female counterparts.

**Table 4:** Binary logistic regression predicting NSI occurrence (N = 188) p < 0.05 = significant (^*^); p < 0.01 = highly significant (^**^) Gender coded as 1 = male and 0 = female NSI, needlestick and sharps injury

Variable	B	SE	Wald	df	p-Value	OR (Exp(B))	95% CI for OR (lower-upper)
Knowledge score	0.199	0.08	6.16	1	0.013^**^	1.22	1.04-1.42
Gender (Male)	0.605	0.302	4.01	1	0.045^*^	1.83	1.01-3.27
Constant	-2.176	1.024	4.52	1	0.033^*^	0.114	-

Model performance statistics are presented in Table 5. Out of the total sample of 188 HCWs, the model explained approximately 9.8% of the variance in NSIs occurrences (Nagelkerke R² = 0.098) and achieved an overall classification accuracy of 70.2% (n = 132/188). This indicates that the model correctly predicted the NSI experience status for the majority of participants. The chi-square test for the overall model showed a significant result, χ²(2, N = 188) = 8.92, p = 0.012, which means the model is useful for telling apart HCWs who have had needlestick injuries from those who have not.

**Table 5:** Model summary statistics for binary logistic regression predicting NSI occurrence (N = 188) p < 0.05 = significant (*) The effect size is interpreted using Nagelkerke R², where a value of 0.098 indicates a small-to-moderate effect. NSI, needlestick and sharps injury

Model summary statistics	Value
Model chi-square (χ²)	8.92
Degree of freedom (df)	2
p-Value	0.012^*^
Effect size (Nagelkerke R²)	0.098 (9.8%)
Overall classification accuracy	70.20%

## Discussion

This study provides meaningful insights into the ongoing risks associated with NSIs among HCWs, revealing that knowledge alone is not sufficient to ensure safe practices. The mean knowledge score of 16.5 ± 2.83 reflects a generally high level of awareness regarding NSI risks and transmission routes. Similar patterns have been reported in studies from Malaysia and the Gulf States, where HCWs demonstrated adequate knowledge levels [17,18]. However, persistent misconceptions, particularly regarding needle recapping and PEP for hepatitis C, remain prevalent. These findings are consistent with earlier research indicating widespread misinformation about safe injection practices and the need for more robust educational interventions [3,10,19].

In terms of attitudes, HCWs showed moderately positive perceptions toward NSI prevention, which aligns with literature that emphasizes the influence of organizational culture and institutional climate on safety perceptions [11,20]. Despite this, the practice score was low (M = 4.10 ± 1.12), suggesting a disconnect between knowledge, attitudes, and actual behavior. Common lapses included inconsistent use of personal protective equipment, improper disposal of sharps, and underreporting of NSIs. These issues may be driven by institutional blame cultures, fear of punitive responses, and inadequate feedback mechanisms [21].

The interrelationship between KAP was further assessed using Pearson’s correlation analysis. A moderate positive correlation between knowledge and attitude (r = 0.305) suggests that increased awareness may improve safety perceptions, consistent with integrative reviews on safety behavior dynamics [18]. However, the weaker correlations between knowledge and practice (r = 0.289) and between attitude and practice (r = 0.264) reveal a persistent “know-do” gap, indicating that information alone does not lead to consistent safe practices [22]. These behavioral gaps are likely influenced by individual, institutional, and systemic barriers, which necessitate tailored interventions that address more than cognitive understanding [18].

Importantly, the data revealed that more than one-third of participants (n = 64, 34.2%) had experienced at least one NSI. This high rate of exposure aligns with international trends, where frequent NSIs are compounded by weak reporting structures and limited post-exposure follow-up. Logistic regression analysis identified two significant predictors of NSI occurrence: higher knowledge scores and male gender. HCWs with greater knowledge were more likely to report NSIs (OR = 1.22, p = 0.013), suggesting that increased awareness may facilitate recognition and reporting of incidents rather than indicating increased risk per se. In addition, male HCWs were nearly twice as likely to report NSIs (OR = 1.83, p = 0.045), echoing findings from studies in Saudi Arabia and Southeast Asia that highlighted gender as a significant predictor of exposure [18]. These disparities may stem from differences in clinical task assignments, gender-related behavioral tendencies, or female HCWs' underreporting or stigma. This points out the need for gender-sensitive research to understand the psychosocial and occupational contexts influencing NSI reporting.

The implications of these findings extend beyond individual education. Interventions must move past awareness campaigns and incorporate structured behavior change strategies, institutional accountability, and policy-level enforcement. For example, the Needlestick Injury Prevention (N-SIP) module developed by Mohd Kutubudin et al. integrates educational content with hands-on skills training and systematic institutional support [16,23]. Embedding such programs into professional development, along with routine audits, could significantly improve adherence and reduce underreporting.

Healthcare systems should also invest in strong reporting infrastructures that support open disclosure and nonpunitive responses to NSIs, fostering a culture of safety and transparency. Ongoing education on updated infection prevention strategies coupled with the use of safety-engineered devices can further protect HCWs from occupational injuries [24,25]. In alignment with WHO’s goal of eliminating preventable NSIs, adapting global safety guidelines into national healthcare policies is essential [17]. These findings collectively support the need for comprehensive reform, balancing individual responsibility with systemic institutional support [26].

This study has several limitations. First, the cross-sectional design restricts causal inference between KAP levels and NSI occurrence. Second, reliance on self-reported data may have introduced recall and social desirability bias, particularly in relation to past injuries. Third, the single-facility setting limits generalizability to other clinical contexts in Oman. Fourth, the absence of the adapted questionnaire items in the manuscript reduces transparency regarding potential modifications from the original instrument, and limited detail on KAP score calculation may affect reproducibility. Additionally, underreporting remains a challenge in NSI research, potentially leading to underestimated incidence despite assurances of confidentiality. Finally, unmeasured variables such as job role, exposure frequency, and organizational safety culture may also influence risk.

Future research should adopt longitudinal and mixed-methods designs to explore behavioral and institutional determinants of NSI prevention in greater depth. Intervention trials evaluating scenario-based training modules, safety-engineered devices, and nonpunitive reporting systems in Oman are particularly warranted. Expanding multi-site studies and ensuring transparency in measurement tools will further strengthen evidence. Such approaches will help determine whether knowledge translates into safer practice, while also guiding culturally appropriate policies and nationwide NSI prevention strategies.

## Conclusions

This study highlights the ongoing risk of NSIs among HCWs, despite generally adequate levels of knowledge. While mean knowledge scores indicated satisfactory awareness, critical misconceptions such as the perceived acceptability of needle recapping and limited understanding of post-exposure procedures remain evident. The finding that nearly one in three HCWs had sustained an NSI underscores the urgency of implementing measures that go beyond knowledge dissemination alone. Although positive correlations were observed between KAP, the persistence of a “know-do” gap demonstrates that awareness does not automatically translate into consistent safe practice. This reinforces the importance of supportive organizational environments and sustainable behavioral engagement strategies.

Gender disparities in NSI reporting also warrant closer attention, with male HCWs nearly twice as likely to report incidents compared with their female counterparts. These differences may be linked to variations in task allocation, occupational roles, workplace safety culture, or underreporting behaviors and highlight the need for further qualitative exploration to better understand the underlying dynamics. The findings carry important implications for occupational safety policy and training frameworks. While causality cannot be established within a cross-sectional design, the results suggest that future interventions may benefit from incorporating behavioral change approaches, routine professional development, and mechanisms to strengthen organizational accountability. Programs such as the N-SIP module illustrate how education combined with skills training and institutional support could be evaluated in future trials. Ultimately, reducing NSI incidence is likely to require a multifaceted, system-level response that integrates ongoing education, accessible and nonpunitive reporting mechanisms, and a workplace culture grounded in safety and transparency. Therefore, there is a need to continue longitudinal and interventional studies to assess the effectiveness and sustainability of these approaches, as well as to tackle contextual barriers in diverse healthcare settings.
